# Changes in the composition of the fecal metabolome and gut microbiota contribute to intervertebral disk degeneration in a rabbit model

**DOI:** 10.1186/s13018-023-04486-x

**Published:** 2024-01-03

**Authors:** Shuai Cheng, Jian Yu, Meiling Cui, Hongmin Su, Yang Cao

**Affiliations:** 1 Suzhou Medical College of Soochow University, No. 1 Shizi Street, Suzhou, 215006 China; 2 Department of Spinal Surgery, Heze Municipal Hospital, Heze, 274031 China; 3 Department of Oncology, Heze Municipal Hospital, Heze, 274031 China; 4 Department of Orthopedics, The First Affiliated Hospital of JinZhou Medical University, No. 2, Section 5, Renmin Street, Jinzhou, 121012 China

**Keywords:** Lower back pain, Intervertebral disk degeneration, Gut microbiota, Fecal metabolomics

## Abstract

**Purpose:**

Lower back pain (LBP), mainly caused by intervertebral disk (IVD) degeneration (IDD), is widely prevalent worldwide and is a serious socioeconomic burden. Numerous factors may trigger this degenerative process, and microbial dysbiosis has recently been implicated as one of the likely causes. However, the exact relationship between IDD and the microbiome remains obscure. In this study, we investigated the gut microbiota composition and fecal metabolic phenotype and discussed the possible influences of microbiome dysbiosis on IDD.

**Methods:**

Fecal DNA was extracted from 16 fecal samples (eight rabbit models with IDD and eight sex- and age-matched healthy controls) and analyzed by high-throughput 16S rDNA sequencing. The fecal samples were also analyzed by liquid chromatography–mass spectrometry-based metabolomics. Multivariate analyses were conducted for the relationship between the omics data and IDD, linear discriminant analysis effect size was employed for biomarker discovery. Moreover, the Kyoto Encyclopedia of Genes and Genomes (KEGG) database was used to annotate the differential metabolites. The potential correlation between differential gut microbiota and metabolites was then assessed.

**Results:**

The 16S rDNA sequencing results showed that the β-diversity of the gut microbiota was significantly different between the IDD and control groups, with distinct abundance levels of dominant genera. Moreover, 59 metabolites were significantly upregulated and 91 were downregulated in IDD rabbits versus the controls. The KEGG enrichment analysis revealed that the top pathways remarkably impacted by IDD were tyrosine metabolism, amino sugar and nucleotide sugar metabolism, benzoate degradation, ABC transporters, ascorbate and aldarate metabolism, pantothenate and CoA biosynthesis, and pyrimidine metabolism. The correlation analysis revealed that DL-tyrosine and *N*-acetylmuramic acid were associated with multiple differential bacterial genera, including *Helicobacter* and *Vibrio*, which may play important roles in the process of IVD degeneration.

**Conclusion:**

Our findings revealed that IDD altered gut microbiota and fecal metabolites in a rabbit model. The correlation analysis of microbiota and metabolome provides a deeper understanding of IDD and its possible etiopathogenesis. These results also provide a direction and theoretical basis for the clinical application of fecal transplantation, probiotics, and other methods to regulate gut microbiota in the treatment of LBP caused by IDD.

## Introduction

Lower back pain (LBP) is a global healthcare concern and a leading cause of disability affecting the daily activities of millions of people worldwide [1]. The direct and indirect costs of LBP are estimated to be $100–$200 billion per year in the USA, and over half of this cost is due to reduced productivity [2]. LBP has many pathological causes, such as age and mechanical stress, as well as genetic factors. One of the most likely causes is intervertebral disk (IVD) degeneration (IDD) [2]. Clinically, IDD treatment mostly involves decompression, fusion, and stabilization [3], and there are currently no effective approaches that interrupt the degenerative process. Therefore, it is essential to identify novel treatment options for IDD that are safe and effective.

Evidence of a microbial role (microbes in and on the human body) in the pathogenesis of many human diseases is rapidly emerging through advances in metagenomic technologies. Inflammation regulation in many clinical conditions, including ankylosing spondylitis, osteoarthritis (OA), rheumatoid arthritis, and septic arthritis, is associated with changes to the microbiome composition of the gastrointestinal system, mouth, and skin [4–9]. It has been proposed that the initiation of IDD and the acceleration of disk degeneration have an infectious etiology. The microbiome can be an important factor in inducing or exacerbating IDD by mediating or altering the internal and external IVD microenvironments. For example, inflammation is implicated in the development of IDD, and one of the putative triggers of inflammation is infection of the IVD by skin bacteria, in particular *Propionibacterium acnes* [10, 11].

The digestive tract is inhabited by a diverse network of bacteria in the gut microbiota. Because these microorganisms are composed of both pathogenic and beneficial bacteria, prosperous symbiosis requires a carefully maintained abundance ratio. Additionally, bacteria can impact the host phenotype by secreting specific microbial metabolites. Reportedly, many chronic diseases associated with musculoskeletal disorders, including autoimmune diseases, cancer, frailty, inflammatory bowel diseases, malnutrition, obesity, and type 2 diabetes, have been tied to the gut microbiota and its adverse perturbations [12, 13]. Traditionally, these conditions have been found to be risk factors of IDD. Three mechanisms reveal how gut microbiota contribute to IDD: (1) modulated nutrient absorption and metabolite formation in the gut and diffusion into the IVD; (2) bacterial translocation across the gastrointestinal epithelial barrier and into the IVD; and (3) mucosal and systemic immune regulation [14]. It has become increasingly recognized that one of the many effects of IDD is noticeable perturbations in gut microbial communities. Current research shows that changes in the composition of the microbiome and associated metabolites may contribute significantly to IDD [15]. However, it remains unclear if microbial changes in the gut microbiota are involved in IDD. The effects of IDD on gut bacteria have also not been characterized.

To investigate the association between IDD and gut microbiota, we developed a rabbit model of IDD and followed an integrated approach using fecal ultra-high-performance liquid chromatography–mass spectrometry (UHPLC–MS) and 16S rDNA gene sequencing. Potential non-surgical strategies to correct IDD can target the gut microbiome to inhibit inflammation and interrupt the amplification of cascade reactions. Because of the feasibility of fecal microbiota transplantation (FMT), further knowledge of the gut microbiota and gut microbiota-derived metabolites in IDD may offer additional insight into LBP prevention, development, and treatment.

## Materials and methods

### Experimental animals

We obtained 16 healthy male New Zealand rabbits from Jiangxi Ganzhou Animal Husbandry Research Institute. The animals were between 4 and 5 months old, and the weight of the animals ranged from 2.5 to 3.0 kg. We gave the animals ordinary food and water ad libitum and maintained suitable light control (12 h light/dark cycle) and temperature conditions (20–26 °C). After the rabbits acclimatized for one week, we divided them randomly into control and IDD groups (*n* = 8/group). To construct the IDD models, we anesthetized the experimental rabbits by slowly injecting 3% sodium pentobarbital (30 mg/kg) through the ear vein, and then performed fibrous ring paracentesis on these rabbits. To confirm that the model was established, we conducted an X-ray examination with a magnetic resonance imaging (MRI) T2WI sequence. The Ethical Management Committee of Heze Municipal Hospital (Approval Number: 2021-KY004) approved the experimental procedures, which we implemented according to relevant regulations and guidance.

### Sample collection

The two groups of experimental rabbits were fed for 4 weeks, and then, fresh feces were collected from all rabbits in the morning on the same day. We placed the fecal samples in sterile centrifuge tubes, froze them in liquid nitrogen, and stored at − 80 °C until sequencing.

At 4 weeks after surgery, the animals in both groups were sacrificed by intravenous injection of an overdose of 3% sodium pentobarbital (100 mg/kg), and the complete intervertebral disk tissues were immediately dissected and removed. After fixation and decalcification, the specimens were cut in the mid-sagittal plane, then dehydrated, embedded in paraffin, and sliced.

### Hematoxylin and eosin staining

Sections of IVD tissue were stained with hematoxylin and eosin (H&E). We first incubated the slides at 60 °C and then soaked them in xylene before dehydrating them in ethanol. Next, we stained the sections with hematoxylin for 5 min and with eosin for 5 min. We washed the slide with distilled water, and then dehydrated them in an ethanol gradient. Finally, we blocked the slides with neutral resin and imaged them using a microscope.

### 16S rDNA sequencing

We used a Magnetic Soil and Stool DNA Kit (TIANGEN Biotech Co., Ltd., Beijing, China) to extract the total genome DNA from the samples. We used 1% agarose gels to determine purity and DNA concentration. We used barcoded primers to amplify 16S rRNA genes. We conducted the polymerase chain reactions (PCR) in 30 μL reactions with 15 μL of Phusion High-Fidelity PCR Master Mix (New England Biolabs, Ipswich, MA, USA). We mixed the PCR products with equal volume of 1 × loading buffer (containing SYBR Green) and conducted electrophoresis on a 2% agarose gel. After we mixed PCR products in equidensity ratios, we purified the mixture with a Qiagen Gel Extraction Kit (QIAGEN, Hilden, Germany). We used the TruSeq DNA PCR-Free Sample Preparation Kit (Illumina, San Diego, CA, USA) to generate the sequencing libraries following the manufacturer’s instructions. We added index codes and assessed library quality on an Agilent Bioanalyzer 2100 system (Agilent Technologies, Santa Clara, CA, USA) and a Qubit 2.0 Fluorometer (ThermoFisher Scientific, Waltham, MA, USA). We used an Illumina NovaSeq6000 platform to sequence the libraries and obtained 250 bp paired-end reads.

### 16S rDNA microbial community analysis

Because some of the reads overlapped reads that were generated from the opposite end of the same DNA fragments, we used FLASH to merge the paired-end reads. We conducted sequence analyses using UPARSE software package with the UPARSE-operational taxonomic unit (OTU) and UPARSE-OTUref algorithms. We then assigned sequences with 97% or higher similarity to the same OTUs. Next, we used the RDP classifier to annotate taxonomic information and obtained representative sequences of selected OTUs (Silva 132 for 16S, UNITE for ITS). After rarifying the OTU table, we computed alpha diversity (α-diversity) by calculating four metrics. We used principal component analysis (PCA) and principal coordinate analysis (PCoA) to determine beta diversity (β-diversity). To verify the differences in the abundance of each taxon between groups, we used the STAMP software. In addition, to quantitatively analyze biomarkers within different groups, we used linear discriminant analysis effect size (LEfSe).

### Extraction and LC–MS analysis of fecal metabolites

To remove proteins from the fecal samples, we mixed thawed samples with four volumes of cold methanol/acetonitrile (1:1, v/v). After we centrifuged the mixture for 20 min (14,000*g*, 4 °C), we obtained the supernatant and dried it in a vacuum centrifuge. We next performed the LC–MS analysis by redissolving the samples in acetonitrile/water (1:1, v/v) and centrifuging the samples for 15 min (14,000*g*, 4 °C). We then subjected the supernatant to the LC–MS analysis.

We used an Agilent 1290 Infinity LC ultra-high-performance liquid chromatography system (UHPLC) (1290 Infinity LC, Agilent Technologies) to perform the chromatographic separations. To collect the first- and second-order spectrum data of the metabolites eluted from the column, we used a Triple TOF 6600 mass spectrometer (AB Sciex Triple TOF 6600), which we operated in the negative and positive ion modes. To evaluate the reliability of the experimental data and monitor the stability of the system, we inserted QC samples in the sample queue.

### Bioinformatic analysis of fecal metabolome data

We completed sum-normalization and processed data using the R package (ropls). We subjected the data to multivariate data analyses, including orthogonal partial least-squares discriminant analysis (OPLS-DA) and Pareto-scaled PCA. To indicate the contribution of each variable to the classification, we calculated variable importance in the projection (VIP) value for each variable in the OPLS-DA model. To determine significance between differences in the two groups of independent samples, we applied Student’s *t* test. To screen for significantly different metabolites, we used a *P* value less than 0.05 and a VIP greater than 1. We also performed a Kyoto Encyclopedia of Genes and Genomes (KEGG) enrichment analysis on the significantly different metabolites.

### Statistical analysis

To analyze the correlation between the metabolites (VIP > 1 and *t* test *P* value < 0.05) and the microbiota (LEfSe LDA > 2 and *P* value < 0.05), we used Spearman’s rank correlation coefficient. We considered a value of *P* < 0.05 to indicate significance in all statistical tests.

## Results

### Establishment of the rabbit IDD model

**Fig. 1:**
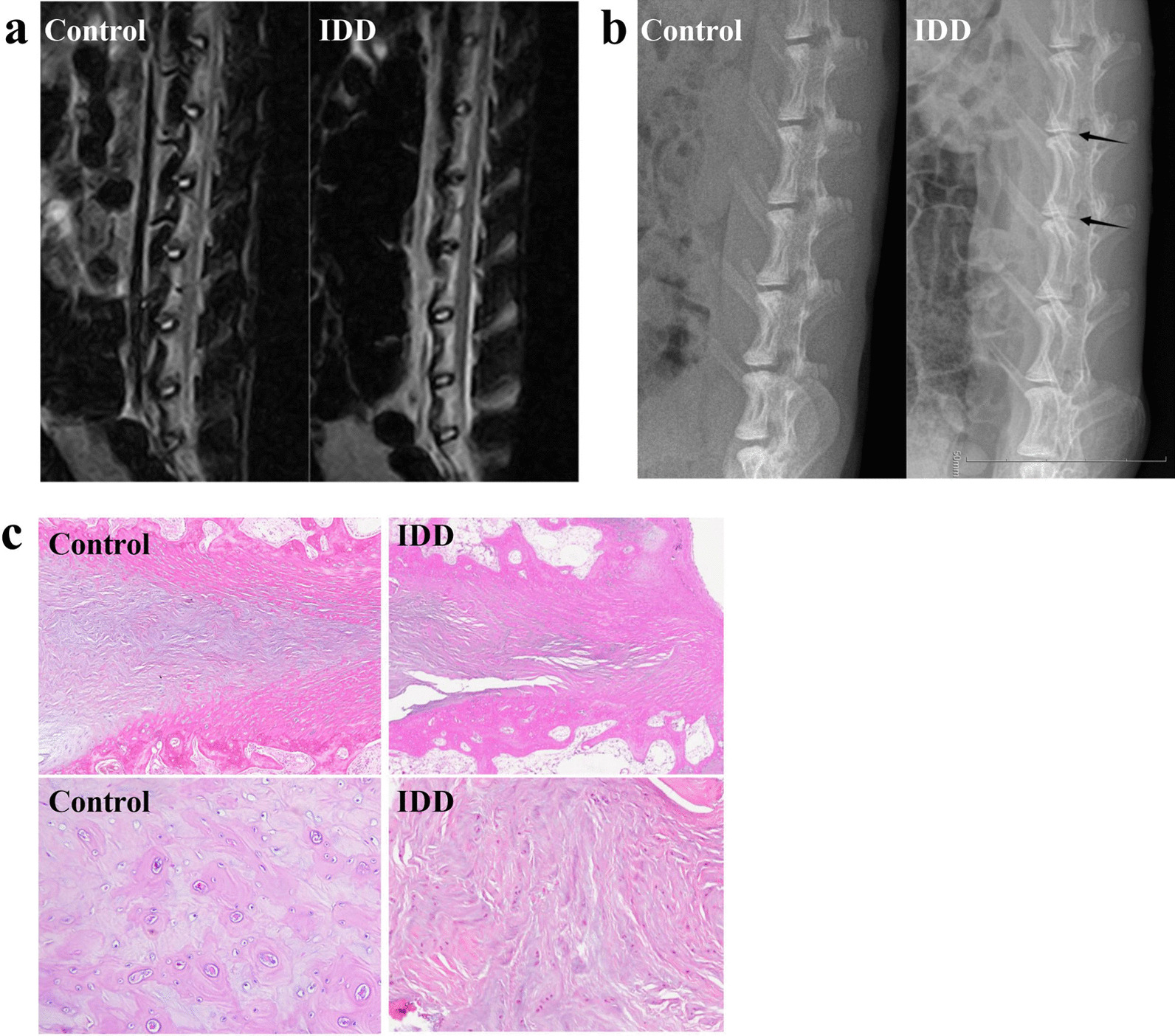
Establishment of the rabbit IDD model. We punctured the disk to induce IDD in rabbits. **a**, **b** MRI T2WI imaging (**a**) and X-ray imaging (**b**) of the IDD model and control groups 4 weeks after the operation. **c** Representative images of hematoxylin and eosin (H&E) staining of the nucleus pulposus from the IDD and control groups

To simulate natural degeneration in rabbits, we punctured the disk. After 4 weeks, we used MRI T2WI imaging to compare results with the control group, and the model met the preset imaging conditions (Fig. 1a). According to X-ray results, the intervertebral space height between the control and model groups was significantly different (Fig. 1b). We also performed histopathological examinations on the IVD tissues to determine changes in the IVD morphology in the IDD rabbits. The H&E staining images from the IDD group exhibited gaps in the nucleus pulposus and a reduction of nucleus pulposus cells (Fig. 1c), while the control group appeared normal. Our results verified that we had successfully induced experimental IDD.

### IDD species abundance of gut microbiota is significantly changed in IDD

We conducted an analysis of microbiome diversity based on 16S rDNA sequencing. To explore whether IDD may be initiated by pathogenic microbes and establish correlations among the presence of certain bacteria with this disease, we clustered the clean reads of all samples in each group. We studied the diversity of the species composition and detected 3716 OTUs by clustering the clean reads at a 97% threshold.

We further calculated seven *α*-diversity indices (Observed species, Shannon, Simpson, Chao1, ACE, Coverage, and PD whole tree; Table 1). No statistically significant difference in these α-diversity indices was observed, indicating no substantial change in the microbial richness and diversity or the total number of OTUs between the IDD and healthy control groups.

**Table 1:** Seven α-diversity indices of gut microbiota in the rabbit IDD models and healthy controls

Rabbit	Shannon	Simpson	Ace	Goods coverage	Chao1	Observed species	PD whole tree
Control 1	7.8482	0.9843	2245.5234	0.9932	2181.9020	1872	107.3642
Control 2	6.7950	0.9640	1261.5514	0.9965	1235.8389	1080	63.4243
Control 3	7.4832	0.9849	1423.9884	0.9960	1394.9133	1215	72.5811
Control 4	7.6733	0.9803	2206.2022	0.9934	2173.6596	1842	106.4973
Control 5	7.8084	0.9772	2453.3568	0.9924	2398.2254	2106	196.1021
Control 6	7.8217	0.9815	2290.9826	0.9929	2267.1628	1965	124.2583
Control 7	7.8373	0.9704	2517.5835	0.9933	2460.6400	2226	126.4508
Control 8	7.2291	0.9707	2198.2458	0.9927	2155.5171	1808	105.3141
IDD1	7.2504	0.9605	2305.0304	0.9928	2249.0667	1911	110.3864
IDD2	7.5601	0.9794	2086.9250	0.9929	2061.7500	1755	139.2959
IDD3	8.1159	0.9875	2449.2170	0.9929	2406.6940	2078	136.5927
IDD4	8.4912	0.9911	2524.7014	0.9926	2473.5430	2163	180.1615
IDD5	7.9243	0.9887	2272.3677	0.9926	2265.3250	1885	135.3511
IDD6	6.4662	0.9424	1302.0239	0.9967	1292.6944	1105	92.2231
IDD7	6.5454	0.9637	1299.4323	0.9960	1285.2000	1086	94.4665
IDD8	7.0642	0.9644	2101.7241	0.9937	2070.6263	1753	127.5300
*P* value	0.6525	0.5189	0.9591	0.8785	0.9591	0.7984	0.4302

**Fig. 2:**
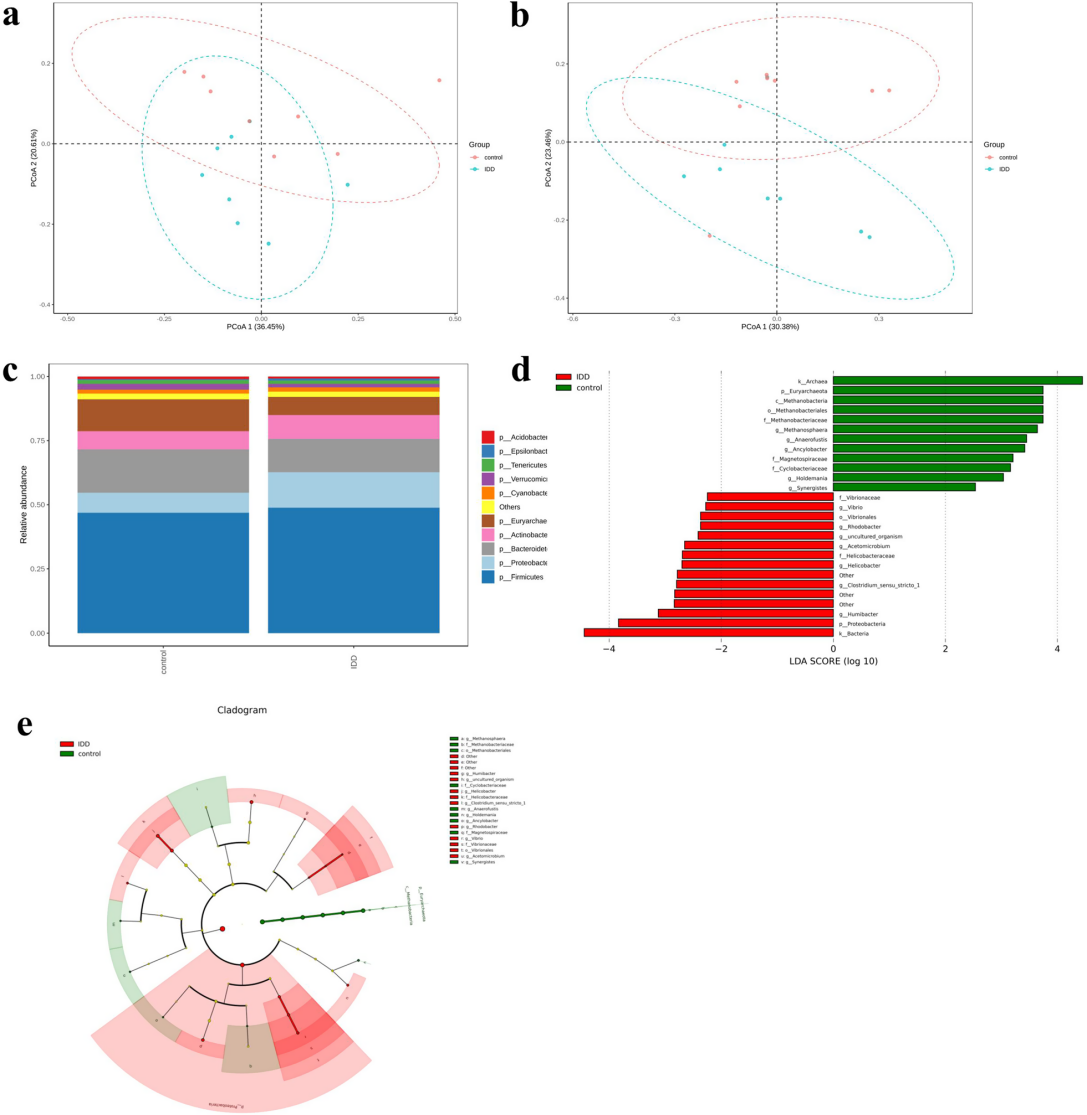
IDD significantly changes the species abundance of gut microbiota. Compositional alteration of gut microbiota between the rabbit IDD models and healthy controls. **a**, **b** PCoA of the weighted (**a**) and unweighted (**b**) UniFrac distances for the normal controls (red circles) and rabbit IDD models (green circles). **c** Relative gut microbiota abundance in the control and IDD groups at the phylum level. **d** LEfSe analysis revealing significant differences in the bacterial taxa of the rabbit IDD models and the normal controls. **e** LEfSe cladogram showing six taxonomic levels from kingdom to genus. Significantly enriched bacterial taxa obtained in healthy controls are indicated by green circles and shading. Significantly enriched bacterial taxa obtained in the rabbit IDD models are indicated by red circles and shading

The composition of bacterial communities can be determined according to β-diversity. According to the PCoA of the weighted UniFrac distance (Fig. 2a) and unweighted UniFrac distance (Fig. 2b), IDD and control sample clusters were separated by relatively far distances. This result suggested that IDD had changed the structure of the gut microbiota.

For the species analysis, we generated a stacked bar chart. The top 10 species with the greatest abundance at the phylum level for each group were selected, and we considered the remaining species to be unclassified (Fig. 2c). The four main phyla (Firmicutes, Proteobacteria, Bacteroidetes, and Actinobacteria) accounted for 80% of the total microbiome. The abundance of Bacteroidetes was lower in the IDD group than in the control group, whereas the abundance of Firmicutes, Proteobacteria, and Actinobacteria was higher.

To study which bacterial taxa revealed differences in the healthy control groups versus the rabbit IDD, we performed LEfSe analysis. According to the results, 12 bacterial taxa were enriched in the healthy controls, and 15 bacterial taxa were enriched in IDD rabbits (LDA scores [log10] > 2 and *P* < 0.05) (Fig. 2d). The cladogram showing the phylogenetic distribution at six different levels from kingdom to genus was obtained using the LEfSe analysis method (Fig. 2e). At the genus level, we observed 11 species with significant differences, of which 6 were upregulated (*Clostridium *sensu stricto* 1* (LDA = 2.80, *P* = 0.014), *Acetomicrobium* (LDA = 2.65, *P* = 0.006), *Helicobacter* (LDA = 2.70, *P* = 0.035), *Humibacter* (LDA = 3.13, *P* = 0.027), *Rhodobacter* (LDA = 2.37, *P* = 0.043), and *Vibrio* (LDA = 2.28, *P* = 0.006)) and 5 were downregulated (*Ancylobacter* (LDA = 3.42, *P* = 0.027), *Synergistes* (LDA = 2.54, *P* = 0.036), *Methanosphaera* (LDA = 3.64, *P* = 0.006), *Holdemania* (LDA = 3.04, *P* = 0.018), and *Anaerofustis* (LDA = 3.45, *P* = 0.011)).

### IDD markedly alters the fecal metabolome

**Fig. 3:**
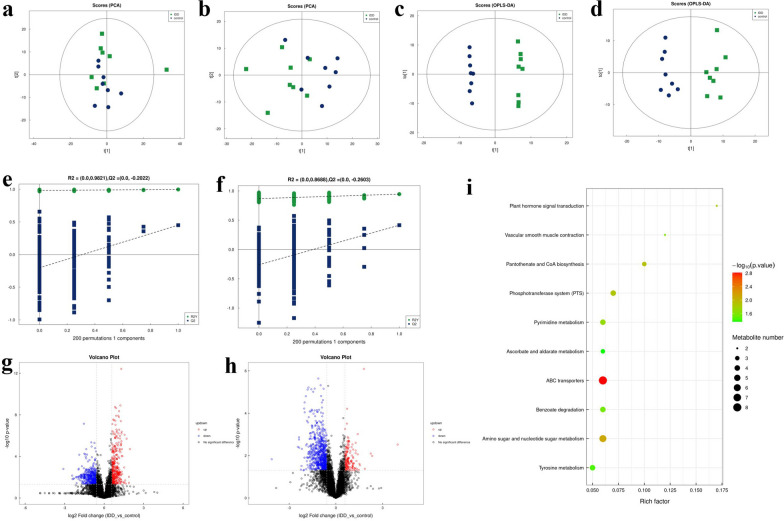
IDD markedly alters the fecal metabolome. LC–MS technology confirmed differences in the IDD and control group metabolic profiles. **a**, **b** Principal component analysis (PCA) score plot of the LC–MS spectra data for the IDD and control groups in positive ion (**a**) and negative ion (**b**) modes. **c**, **d** Orthogonal partial least-squares discriminant analysis (OPLS-DA) score plot of the LC–MS spectra data in positive ion (**c**) and negative ion (**d**) modes. **e**, **f** OPLS-DA permutation testing showing response of 200 permutations in the positive (**e**) and negative (**f**) ion modes. **g**, **h** The volcano charts showing the differences in positive ion metabolites (**g**) and negative ion metabolites (**h**) between the groups. Red dots (up) represent significantly upregulated metabolites (OPLS-DA VIP > 1, *P* < 0.05); green dots (down) represent significantly downregulated metabolites (OPLS-DA VIP > 1, *P* < 0.05); black dots (no significant difference) represent insignificantly changed metabolites. **i** Bubble diagram of the 10 most enriched KEGG pathways in the comparison of the IDD and control. The sizes of the bubbles indicate the metabolite numbers enriched in the KEGG pathways, and the color of the bubble represents the *P*-value

To assess whether IDD disrupted the rabbit metabolome, we conducted an LC–MS analysis of the fecal metabolites. Screening of differential metabolites was first executed by multivariate analysis. To reveal the global metabolic changes in control and IDD rabbits, we used PCA analysis. According to the results, both negative and positive ion modes distinguished the data points of the two groups, which indicated distinct concentrations, quantities, and types of metabolites (Fig. 3a, b). According to the OPLS-DA model, we identified significant differences in the metabolic phenotype between the control and IDD models. The established OPLS-DA model had good reproducibility (R2Y: 0.997 for positive ion, 0.945 for negative ion) and good predictability (Q2: 0.450 for positive ion, 0.415 for negative ion), suggesting that a distinct metabolic profile might exist in the rabbit IDD models (Fig. 3c, d). Additionally, we performed 200 random permutations on the OPLS-DA model to ensure its effectiveness (Fig. 3e, f). In positive ion mode, *R*^2^ = (0.0, 0.9821) and *Q*^2^ = (0.0, − 0.2022); in negative ion mode, *R*^2^ = (0.0, 0.8688) and *Q*^2^ = (0.0, − 0.2603). The original values on the right were higher than all *R*^2^s and *Q*^2^s. According to the data results, the fitting was valid. The original model proved the reliability of the sample data.

A volcano chart was generated to identify possible gut biomarkers of the variation tendencies (Fig. 3g, h). In the IDD rabbits versus the healthy controls, 91 metabolites were considerably decreased and 59 metabolites were significantly increased (OPLS-DA VIP > 1 and *P* value < 0.05). We used the KEGG database to annotate the differential metabolites and found that 97 key metabolites participated in the signaling pathways (Table 2). We also conducted a KEGG enrichment analysis of the differential metabolites. Figure 3i shows the top 10 metabolites between the two groups. Among the metabolic pathways, we assessed primarily amino acid metabolism (tyrosine metabolism) and carbohydrate metabolism (amino sugar and nucleotide sugar metabolism). Four differential metabolites were enriched in the tyrosine metabolism pathway, namely, DL-tyrosine, *p*-coumaric acid, salidroside, and pyruvate; 6 were enriched in the amino sugar and nucleotide sugar metabolism pathway (*N*-acetylmuramic acid, *D*-arabinose, *D*-galacturonic acid, *N*-acetyl-d-mannosamine, n-acetylneuraminic acid, and *N*-acetyl-d-glucosamine; Table 3).

**Table 2:** Annotation results of the differential metabolites using the KEGG database

Metabolite	KEGG ID
Dl-lactate	C01432
Thymine	C00178
Trans-ferulic acid	C01494
3-Hydroxy-4-methoxycinnamic acid	C10470
Shikimate	C00493
Xanthine	C00385
*N*-acetylmuramic acid	C02713
DL-tyrosine	C00082
*D*-arabinose	C00259
*P*-coumaric acid	C00811
*D*-turanose	C19636
4-Pyridoxic acid	C00847
Alpha-ketocaproic acid	C00902
Salidroside	C06046
Prostaglandin i2	C01312
Pyruvate	C00022
Pomiferin	C10519
*D*-mannose	C00936
Ile-Pro	C00526
Teniposide	C11153
Uracil	C00106
*D*-galacturonic acid	C00333
Atorvastatin	C06834
*N*-acetyl-d-mannosamine	C00645
*D*-lactose	C00243
16-Hydroxyhexadecanoic acid	C18218
Pantothenate	C00864
Cis,cis-muconic acid	C02480
Benzoic acid	C00180
Deoxyinosine	C05512
*N*-acetylneuraminic acid	C00270
4-Hydroxybenzoate	C00156
Jasmonic acid	C08491
Enterodiol	C18166
Eurycomalactone	C08759
Apigenin	C01477
Phenyllactic acid	C01479
Oryzalin	C18877
Butanoic acid	C00246
Dicyclomine	C06951
Tomatidin	C10826
Bisoprolol	C06852
Trilostane	C12580
Furmecyclox	C18912
Cabergoline	C08187
Fenpropimorph	C18787
20-Hydroxyarachidonic acid	C14748
Thioperamide	C17933
Tetrabenazine	C11168
Orphenadrine	C07935
N6-methyladenine	C08434
Tamoxifen	C07108
Penbutolol	C07416
Dimethametryn	C18537
Eupatilin	C10040
Debrisoquin	C13650
Karakoline	C08693
Dendrobine	C09943
Oxandrolone	C07346
Ginkgolic acid i	C10794
*L*-palmitoylcarnitine	C02990
Leucylleucine	C11332
Nivalenol	C06080
Convolvamine	C10854
Nicotinamide	C00153
Lsd	C00715
Bupirimate	C18776
Paroxetine	C07415
2′-Deoxycytidine	C00881
Dihydrocapsaicin	C16952
Tetramethrin	C18373
5-Methyl-2′-deoxycytidine	C03592
Cytarabine	C02961
Guanine	C00242
5-nitro-2-(3-phenylpropylamino)benzoic acid	C13705
Nigerose	C01518
N-3-oxododecanoyl-l-homoserine lactone	C21201
Indoleacetic acid	C00954
*N*-acetylglucosamine	C00140
Glucosamine	C08349
*N*-Acetylmannosamine	C00645
Piperlongumine	C10166
Ergonovine	C07543
Ala-Phe	C07375
Isophorone	C14743
Nalidixic acid	C05079
N.epsilon.-methyl-l-lysine	C02728
Lactulose	C07064
*N*-acetyl-d-glucosamine	C00140
2-Aminoethylphosphonic acid	C03557
Glycitein	C14536
5-Aminovaleric acid	C00431
Creatine	C00300
4-(4-aminophenoxy)aniline	C14759
Naltrexone	C07253
Cinobufagin	C16931
Oxethazaine	C12552

**Table 3:** The main metabolic pathways involving the differential metabolites

KEGG pathway	Pathway hierarchy	Metabolite
ABC transporters	Membrane transport	2-aminoethylphosphonic acid, 2′-deoxycytidine, *D*-arabinose, *D*-galacturonic acid, D-lactose, Deoxyinosine, Ile-Pro, *N*-acetyl-d-glucosamine
Amino sugar and nucleotide sugar metabolism	Carbohydrate metabolism	*D*-arabinose, *D*-galacturonic acid, *N*-acetyl-d-glucosamine, *N*-acetyl-d-mannosamine, *N*-acetylmuramic acid, *N*-acetylneuraminic acid
Pantothenate and CoA biosynthesis	Metabolism of cofactors and vitamins	Pantothenate, Pyruvate, Uracil
Phosphotransferase system (PTS)	Membrane transport	*D*-lactose, *N*-acetyl-D-glucosamine, *N*-acetylmuramic acid, Pyruvate
Plant hormone signal transduction	Signal transduction	Jasmonic acid, Indoleacetic acid
Pyrimidine metabolism	Nucleotide metabolism	2'-deoxycytidine, Ile-Pro, Thymine, Uracil
Vascular smooth muscle contraction	Circulatory system	Prostaglandin i2, 20-hydroxyarachidonic acid
Benzoate degradation	Xenobiotics biodegradation and metabolism	4-hydroxybenzoate, Benzoic acid, Cis,cis-muconic acid, Pyruvate
Tyrosine metabolism	Amino acid metabolism	DL-tyrosine, *P*-coumaric acid, Pyruvate, Salidroside
Ascorbate and aldarate metabolism	Carbohydrate metabolism	*D*-arabinose, *D*-galacturonic acid, Pyruvate

### Relationship between differential gut microbiota and fecal metabolome in IDD

**Fig. 4:**
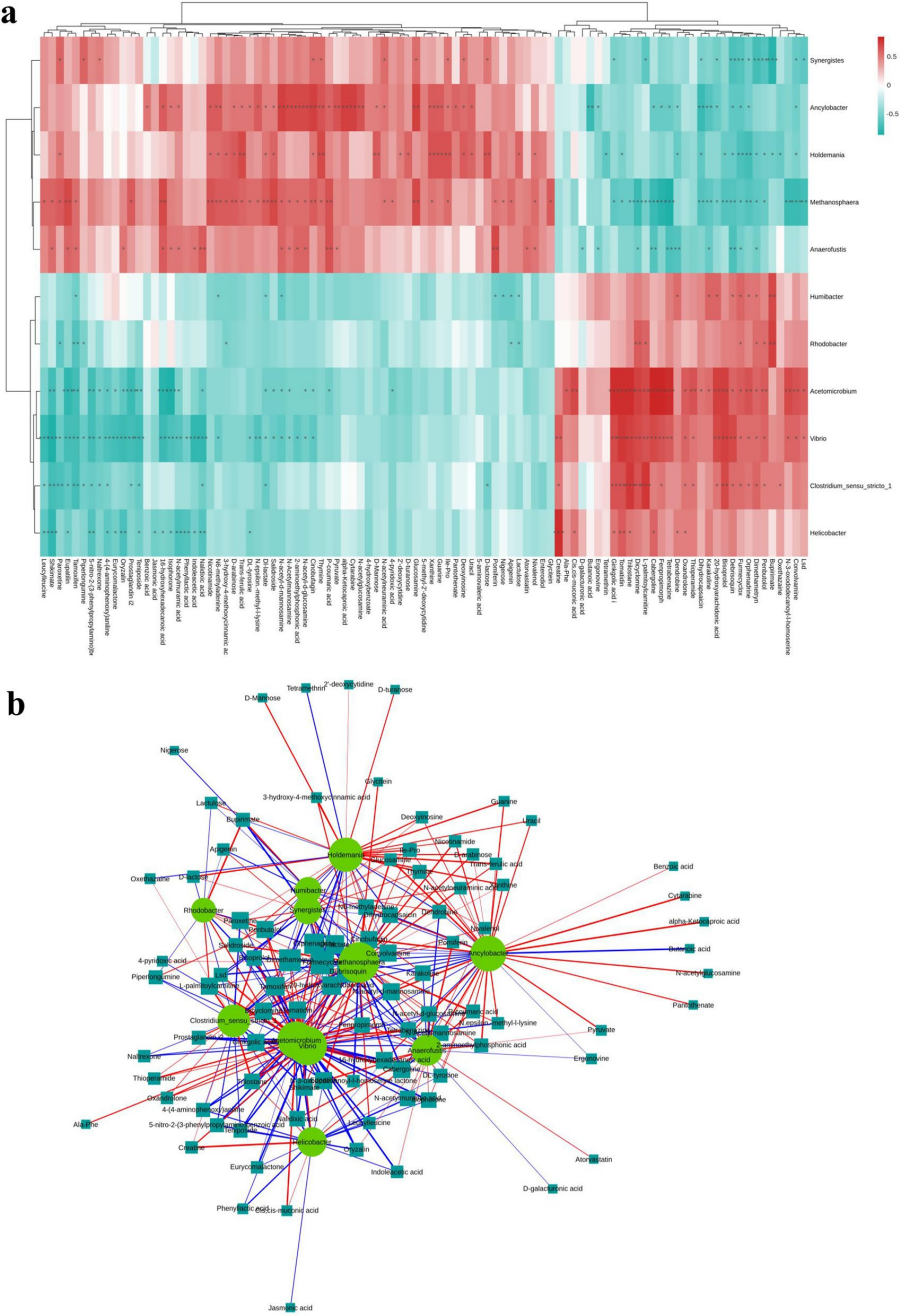
Association analysis of differential gut microbiota and fecal metabolome in IDD. Correlation relationships between discriminative genus-level microorganisms and metabolites in the fecal samples of IDD. **a** Hierarchical clustering heat map of the Spearman correlation analysis of differential gut microbiota and fecal metabolome. **b** Network diagram of Spearman correlation analysis of differential gut microbiota and fecal metabolome. The discriminative genera are marked with circles, and the discriminative fecal metabolites are marked with squares

We next conducted a correlation analysis in the rabbit IDD model between 11 bacterial genera and 97 differential metabolites (Fig. 4a, b). In total, 191 bacteria–metabolite pairs with significant positive correlation and 161 with significant negative correlation were found. The genera correlated with the greatest number of differential metabolites were *Acetomicrobium*, *Ancylobacter*, *Methanosphaera*, and *Vibrio*, which were significantly correlated with 46, 43, 52, and 50 metabolites, respectively. Furmecyclox, debrisoquin, and dimethametryn were most significantly correlated with the gut microbiota and significantly related to 11, 9, and 9 different bacterial genera, respectively. According to our findings, these distinguishing metabolites were quite similar to variations in intestinal microbiota and these discriminative metabolites and gut bacteria were closely related to IDD. We next should determine whether the associated intestinal microbes directly produce these metabolites.

## Discussion

Gut microbiota are indispensable in host physiology, including efficient cellular metabolism, homeostasis, immune system modulation, and nutrient absorption [16]. Therefore, dysbiosis (i.e., imbalances in the gut microbiota) can trigger aberrant immune responses. These disruptions of the host’s local and systemic homeostasis contribute to various disorders [17]. Secondary to immunomodulatory responses are the effects of the gut microbiota and their metabolites in bones. According to research, perturbations in gut microbiota can cause skeletal deterioration in pathophysiological states [18–20]. The correlation between the host metabolites and intestinal microorganisms in IDD has not been extensively investigated. Therefore, we followed a multiomic correlation network approach to analyze these characteristics and to determine the relationship between the fecal metabolome and the microbiome in IDD.

We conducted a 16S rDNA gene sequencing analysis and found that IDD was, in fact, associated with gut microbiota dysbiosis. The α-diversity between the two groups was similar, which demonstrated unaltered richness and evenness. We observed, however, significant differences in *β*-diversity between the control and IDD groups based on the unweighted and weighted UniFrac distances. These structure and quantity changes in intestinal bacteria identified were unique to IDD models and were not observed in control animals on the same dietary regimen. Our findings align with recent observations, showing that gut microbiota dysbiosis may be associated with IDD [14]. Rajasekaran et al. [21] found Firmicutes, Proteobacteria, and Actinobacteria to be abundant in normal human intervertebral disk samples and correlated with antibacterial protection and intestinal barrier function. We did not find a similar pattern at the phylum level; however, the levels of two genera in Firmicutes (*Holdemania* and *Anaerofustis*) and one genus in Proteobacteria (*Ancylobacter*) were lower in the IDD group than in the control group. The differences could be due to the different sample origins. Rajasekaran et al. used intervertebral disks, whereas we used stool samples. In addition, the composition of gut microbiota may vary considerably across individuals based on geography, host genes, age, and other factors, which can lead to diverse connections in studies of intestinal microbiota disorders and degenerative spinal diseases. Thus, it would be intriguing to further establish the disk and skin microbiomes and compare the common or divergent bacteria to the gut microbiota for the evidence of the gut/skin/spine microbiome axis. Clinical testing has uncovered ~ 60 overlapping bacterial strains between the intestine and the IVD [22]. It also remains to be explored if regulating the intestinal microbiota can impact the diversity and abundance of IVD microbiota and alter IDD.

A relationship between gut microbial dysbiosis and degenerative diseases has been suggested [23]. According to a large cross-sectional analysis, numerous gut microbiota features were associated with osteoarthritis (OA), the most common form of arthritis [24]. Notably, the presence of *Lentisphaeria* in abundance was associated negatively with the prevalence of OA and rheumatoid arthritis. Our findings were not similar; however, although IDD and OA are both degenerative diseases, IDD pathogenesis may differ from that of OA.

Firmicutes, Bacteroidetes, and Actinobacteria can digest carbohydrates, such as complex oligoglycans present in mucin [25]. The degradation of these carbohydrates produces short-chain fatty acids, which are reabsorbed by the host for energy [26, 27]. We examined whether there were alterations in gut microbiota metabolites in the rabbit IDD model. The functional annotation of KEGG pathways revealed 97 key altered metabolites in IDD. These differential metabolites were mainly involved in processes such as tyrosine metabolism and amino sugar and nucleotide sugar metabolism. Tyrosine metabolism has been widely confirmed to be closely related to liver disease [28, 29], primary headache and other neurological diseases [30, 31], and it also plays an important role in gut microbiota and bone homeostasis. Dietary phenylalanine, which is caused by the enzyme phenylalanine hydroxylase found in large amounts in the liver, can form tyrosine in mammals. Phenylalanine plays an important role in bone metabolism [32] and is associated with osteoporosis [33]. Phenylalanine and tyrosine are sugar metabolism-regulating substances [34]. Decreased conversion of sugar and energy utilization efficiency has been associated with decreases in DL-tyrosine and other metabolites related to tyrosine metabolism, which are tied to IVD degeneration. Amino and nucleotide sugars are essential to convert carbohydrates and utilize energy [35]. The synthesis of amino and nucleotide sugars begins with the phosphorylation of *N*-acetylmuramic acid through its transport from the periplasmic space to the cytoplasm. Notably, *N*-acetylmuramic acid and other related metabolites were downregulated in the IDD group.

It is possible to identify the bacterial genera that may be related to host metabolic health using correlation analysis [14]. Therefore, to better understand cross talk between the discriminative metabolites and microbiota in IDD, we conducted a correlation analysis. The results showed that *N*-acetylmuramic acid and DL-tyrosine were associated with differential bacterial genera, including *Helicobacter* and *Vibrio*. These bacterial genera are closely related to bone metabolism and were significantly different in the control and IDD groups. *Helicobacter pylori* infection likely induces a chronic systemic inflammatory response, which has been tied to endocrine disorders [36, 37]. Another study showed an association between *H. pylori* and significantly decreased mean lumbar bone mineral density (BMD), which was greatest among men above 50 years old [38]. Reportedly, the prevalence of Proteobacteria increases the incidence of inflammation, metabolic disease, and microbial dysbiosis [39]. Our findings indicate that IDD is associated with certain Proteobacteria. Additional research is needed, however, to determine the potential detrimental impact of Proteobacteria and the underlying mechanisms in bone degeneration disease. *Vibrio parahaemolyticus*, a common pathogen in humans, produces virulence factors that alter the homeostasis and integrity of human systems. Virulent *V. parahaemolyticus* strains cause acute gastroenteritis as well as other distinct diseases [40]. Intestinal inflammation can cause intestinal permeability, which can translocate bacteria as well as toxic metabolites [41], migrating to and gathering near IVDs and thus inducing IDD.

Rodent model studies have shown that spinal cord injury-induced bacterial imbalance in the gut can aggravate the damage and impair recovery [25]. Microbiome dysbiosis represents an imbalance between beneficial and harmful microbes, which can result from various factors, such as diet, disease, and medical interventions [14]. According to our results, IDD regulated numerous bacterial communities, which disrupted the diversity of gut microorganisms. However, the mechanisms by which pathogenic gut bacteria may damage the IVD or beneficial gut bacteria may be protective require further clarification. Several studies in animal models and humans have demonstrated that persistent dysbiosis can modulate peripheral immune cells and the secretion of inflammatory molecules, such as TNF-*α* and IL-1*β* [16]. Notably, these inflammatory cytokines have been reported to be associated with IDD. Therefore, it would be intriguing to examine the host responses to microbiota and if IDD can regulate the gut immune system or systemic inflammation. Additionally, diversities in eukaryotic viruses and prokaryotic phages have been observed in humans [6], and further elucidation of the IDD-associated microbiome may shed light on microbial ecology in the gut and IVD of LBP patients. Intriguingly, FMT was recently performed in a Sprague–Dawley rat IDD model by gavage with fecal bacterial solution and increased the intestinal microbial diversity and abundance, reversed the IDD modeling, and ameliorated the damage to IVD tissue [22]. Increasing evidence suggests that the impairment of some gut microbiota and the metabolite composition can be used as a biomarker for identifying disease progression. Thus, gut microbiota transplantation is promising as a potential therapeutic approach for improving bone dysfunction or reversing pathological conditions.

Despite our findings, our study had several limitations. The experiment only observed the changes in intestinal flora between experimental animals with significant intervertebral disk degeneration and normal controls, while intervertebral disk degeneration is a gradual development process. In future studies, we need to obtain a dynamic view of the intestinal microbiota changes during the process from mild to severe intervertebral disk degeneration. Although we identified correlations among IDD, gut microbiota, and fecal metabolites, we did not clarify their regulatory or causal relationships. Rigorous biomarker studies require adequate sample sizes, but our sample size was small. In the future, studies to identify potential biomarkers for IDD should employ larger sample sizes. In-depth investigations and mechanistic studies are warranted.

## Conclusion

Our study characterized microbiome dysbiosis evidenced by different bacterial community ratios in a rabbit IDD model. We demonstrated for the first time the close correlations among gut microbiota, fecal metabolites, and IDD through a multiomics network approach. According to our results, key factors that affected IVD homeostasis include alterations in the function and abundance of gut microbiota. Insight from these findings revealed the etiopathogenesis of IDD and demonstrated the possibilities of new avenues for non-surgical treatment. Ultimately, this research will improve diagnostic and therapeutic options for patients with lower back pain.

## Data Availability

The datasets used and analyzed during this study are available from the corresponding author upon reasonable request.
